# Supplementary figures and images for: Susceptibility to experimental infection of the invertebrate locusts (Schistocerca gregaria) with the apicomplexan parasite Neospora caninum
Source: PeerJ. 2014 Dec 2;2:e674. doi: 10.7717/peerj.674 (PMC4260130; doi:10.7717/peerj.674)

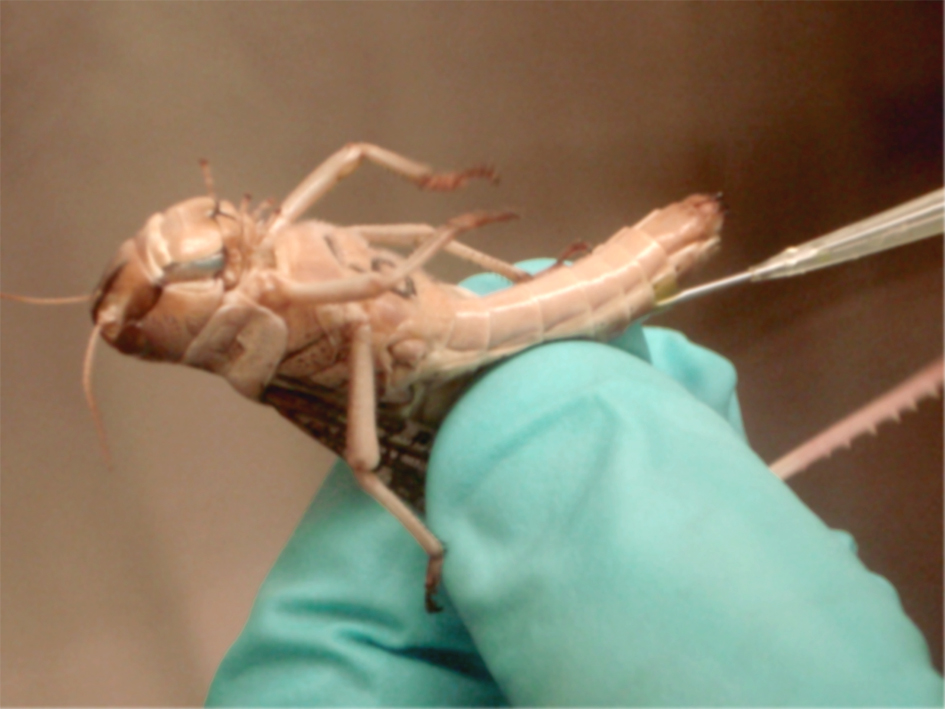

Supplement: Figure S1 [file peerj-02-674-s001.jpg]

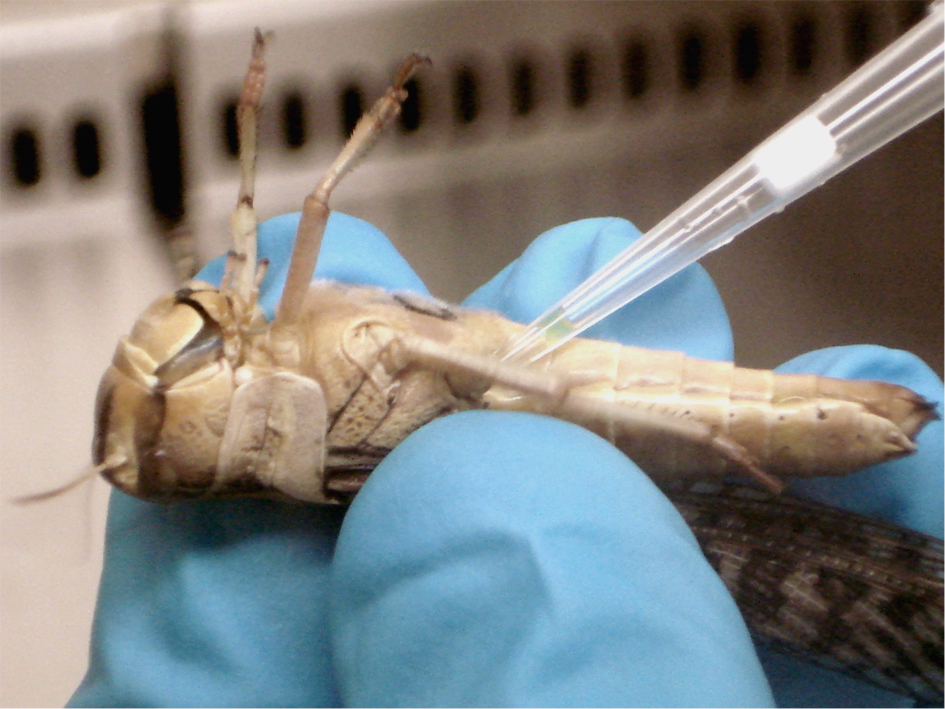

Supplement: Figure S2 — Hemolymph was collected by insertion of a pipette tip through the locust arthrodial membrane at the base of the walking appendages. [file peerj-02-674-s002.jpg]

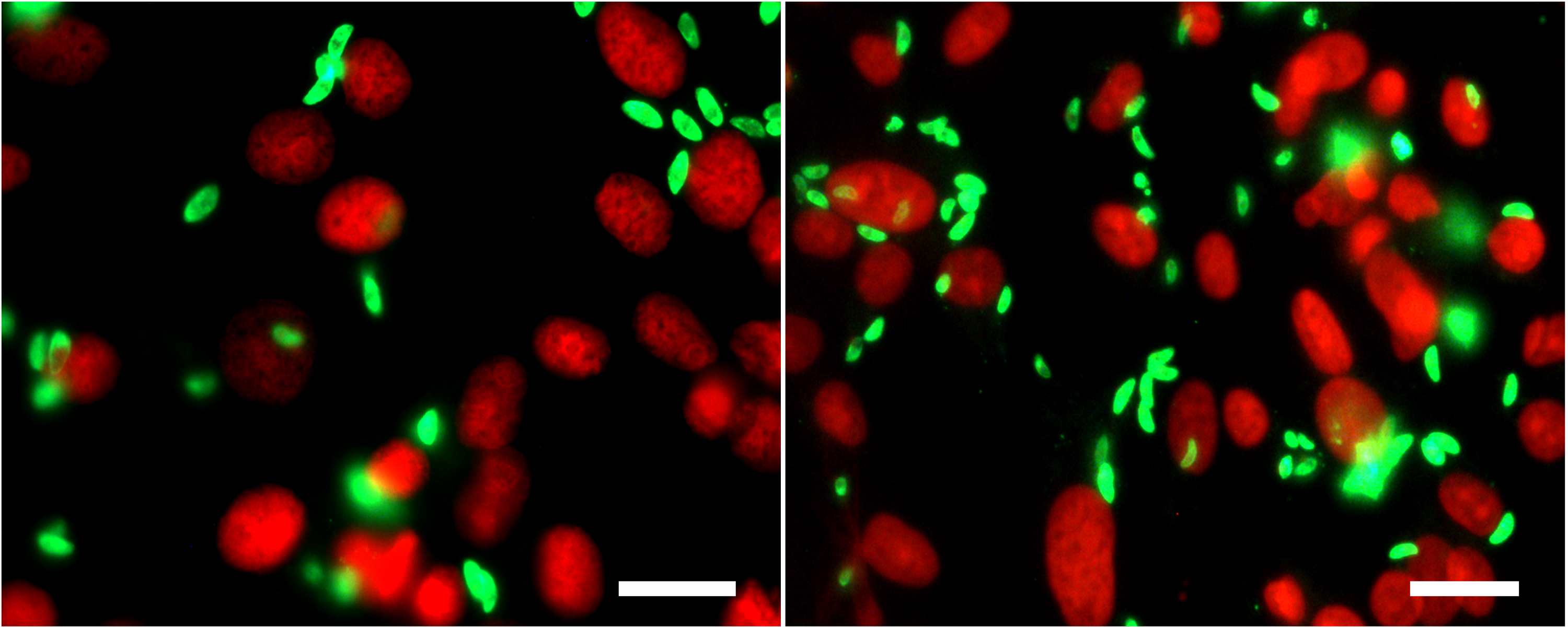

Supplement: Figure S3 — Immunofluorescence micrographs of human brain microvascular endothelial cells (HBMECs) 4 hrs after infection with N. caninum strain isolated from locust brain (A) or original strain (B). Infected HBMECs are immune-labelled with primary monoclonal mouse anti-NcSAG1 antibody that recognizes surface antigen of the parasite and secondary goat-anti-mouse IgG FITC conjugate (green) and nuclear DNA stained with propidium iodide (red). Scale bars, 10 µm. [file peerj-02-674-s003.jpg]

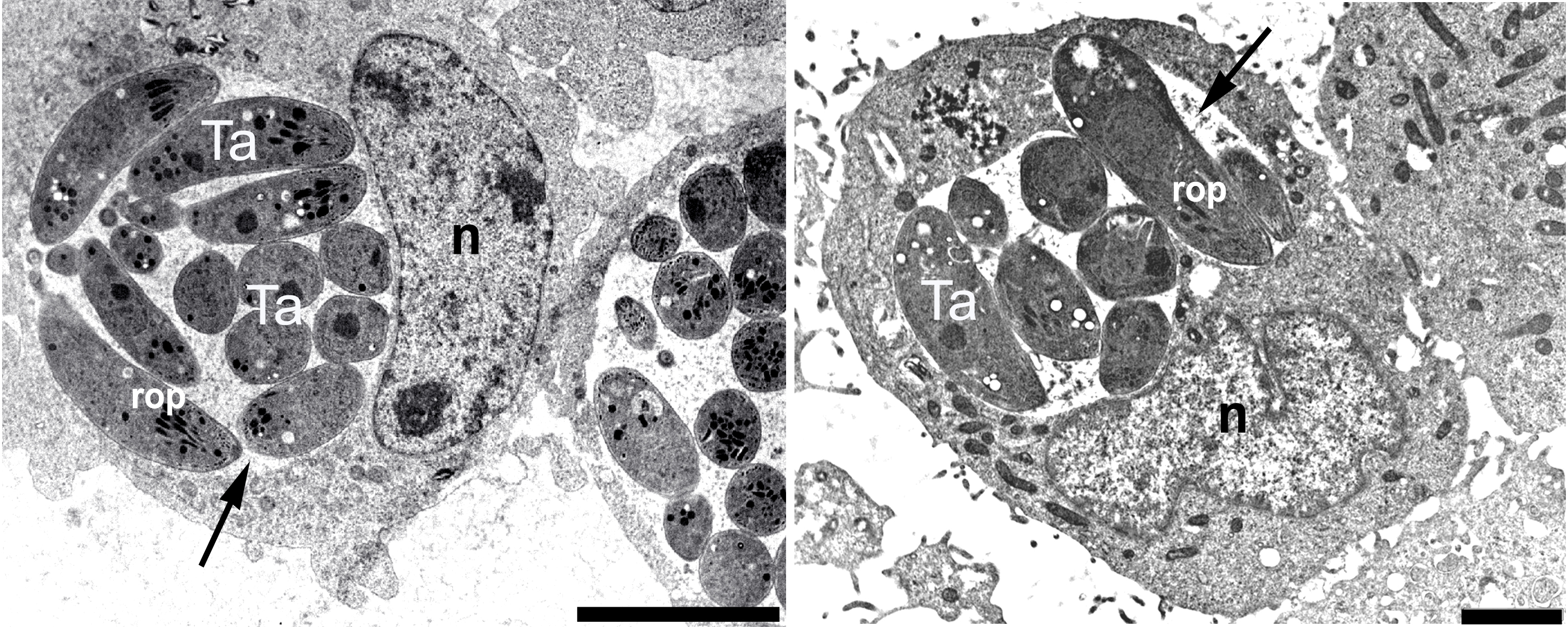

Supplement: Figure S4 — TEM of human brain microvascular endothelial cells (HBMECs) 24 h after infection with N. caninum strain isolated from locust brain (A) or original strain (B). Abbreviations: host cell nucleus (n) and rhoptries (rop). Arrows points at parasitophorous vacuole, which encloses a number of tachyzoites (Ta). Bars in A = 3 µm and b = 2 µm. [file peerj-02-674-s004.jpg]

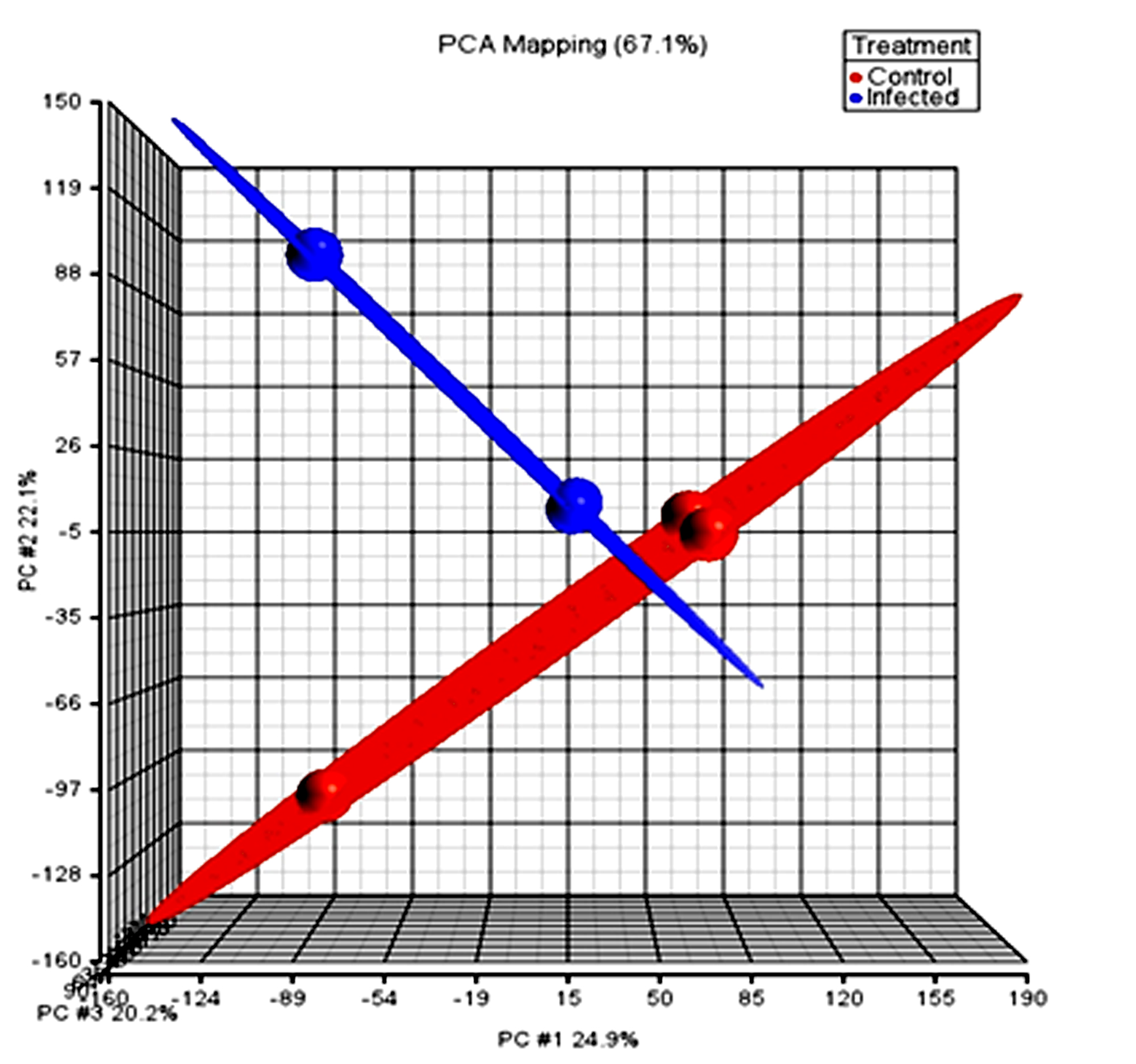

Supplement: Figure S5 — PCA resulted in two relatively distinct components of three infected (blue) and three uninfected samples (red). [file peerj-02-674-s005.jpg]

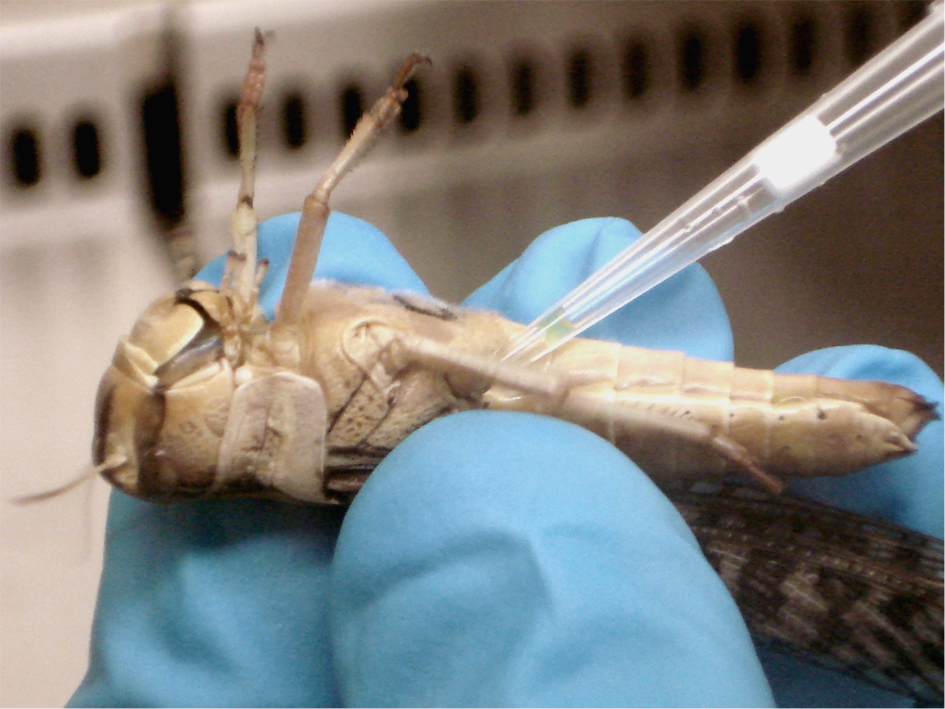

Supplement: Supplemental Information 1 [file peerj-02-674-s007.jpg]
