# Supplementary material for: Susceptibility to experimental infection of the invertebrate locusts (Schistocerca gregaria) with the apicomplexan parasite Neospora caninum
Source: PeerJ. 2014 Dec 2;2:e674. doi: 10.7717/peerj.674 (PMC4260130; doi:10.7717/peerj.674)
Supplement: Table S1 [file peerj-02-674-s006.docx]

**Table S1** Prediction capacity of the multi-variate prediction models trained from the lipid profiles of the infected and control locust brains. Accuracy of each model was estimated using leave-one-out cross-validation

| Lipids used in model | Accuracy | Misclassified samples |
| --- | --- | --- |
| 7: C14:0, C16:0, C16:1, C18:0, C18:1n9c, C18:2n6c and C18:3n3 | 98% | 1 control sample of day 4 predicted as infected |
| 4: C16:0, C16:1, C18:2n6c and C18:3n3 | 98% | 1 control sample of day 4 predicted as infected |
| 3: C16:1, C18:2n6c and C18:3n3 | 98% | 1 control sample of day 4 predicted as infected |
| 2: C16:1 and C18:2n6c | 94% | 1 control sample of day 4 predicted as infected; 1 infected sample of day 1 predicted as control; 1 infected sample of day 5 predicted as control |
